# Understanding and Overcoming Antibody‐Drug Conjugate Resistance: Biological Mechanisms and Emerging Analytical Frameworks in Breast Cancer

**DOI:** 10.1002/advs.202515498

**Published:** 2026-03-24

**Authors:** Minji Seo, Jangsoon Lee, Naoto T. Ueno

**Affiliations:** ^1^ Preclinical Core, Cancer Biology Program University of Hawaiʻi Cancer Center Honolulu Hawaii USA; ^2^ Translational Clinical Research University of Hawaiʻi Cancer Center Honolulu Hawaiʻi USA

**Keywords:** antibody‐drug conjugate, breast cancer, resistance, tumor microenvironment

## Abstract

Antibody‐drug conjugates (ADCs) have revolutionized the treatment landscape of breast cancer by combining the precision of monoclonal antibodies with the potency of cytotoxic agents. Despite the clinical success of ADCs—with 4 FDA‐approved agents to date, and 15 for the entire cancer landscape—their therapeutic durability is frequently undermined by acquired resistance. Rather than arising solely from tumor‐intrinsic alterations, ADC resistance reflects a multi‐layered process shaped by dynamic interactions among cancer cells and the tumor microenvironment (TME), with activation of adaptive signaling networks. For example, stromal architecture, vascular heterogeneity, and immune modulation intersect with clonal evolution, phenotypic plasticity, and pathway reprogramming, thereby constraining ADC delivery and activity. Cutting‐edge technologies such as spatial omics, single‐cell profiling, functional genomics, and patient‐derived models are redefining how these resistance mechanisms are mapped and understood in situ. Building on these insights, emerging therapeutic strategies aim to overcome resistance through mechanism‐guided interventions, including next‐generation ADC designs, co‐targeting of compensatory signaling pathways, and biomarker‐informed therapeutic strategies. Together, these integrated biological and technological perspectives provide a framework for developing more durable and precisely tailored ADC‐based therapies in breast cancer.

## Graphical abstract

Antibody–drug conjugates (ADCs) transform breast cancer therapy, yet resistance limits their durability. Emerging evidence reveals that ADC failure is not solely tumor‐intrinsic but shaped by dynamic tumor–microenvironment interactions that alter drug delivery, processing, and response. Integrating spatial, single‐cell, and functional approaches enables mechanism‐guided strategies to overcome resistance and design more precise, durable ADC therapies.

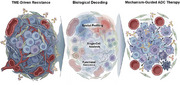

## 1 Introduction

ADCs represent a “smart bomb” strategy for targeted chemotherapy, in which a tumor‐specific monoclonal antibody is conjugated to a highly potent cytotoxic payload. By exploiting tumor‐associated antigens, ADCs enable the selective delivery of the drug to malignant cells while minimizing off‐target toxicity, thereby expanding the therapeutic window compared to conventional chemotherapy. This concept, originally envisioned by Paul Ehrlich's “magic bullet” theory, was realized with the approval of the first ADC in 2000 [1]. In breast cancer, the development of ADCs was propelled by the success of HER2‐targeted antibody therapies and the realization that conjugating ultrapotent cytotoxic agents to these antibodies could help overcome resistance to conventional treatments [2].

As of April 2025, the U.S. FDA has approved 15 ADCs to treat various solid tumors and hematologic malignancies, of which four are widely used to manage breast cancer. Trastuzumab emtansine (T‐DM1, Kadcyla) was the first ADC approved for breast cancer in 2013, following the EMILIA trial, which showed it significantly improved progression‐free survival (PFS) and overall survival (OS) in HER2‐positive metastatic patients compared with lapatinib plus capecitabine [3]. Building on the success of T‐DM1, the next‐generation HER2 ADC, trastuzumab deruxtecan (T‐DXd, Enhertu), was developed using a cleavable linker that enables a bystander effect, allowing the released payload to diffuse and kill neighboring tumor cells regardless of their HER2 expression. Clinically, T‐DXd demonstrated remarkable efficacy in HER2‐positive metastatic breast cancer, achieving a fourfold increase in median PFS compared to T‐DM1 in a head‐to‐head trial (28.8 vs. 6.8 months) [4]. Sacituzumab govitecan (SG, Trodelvy), approved in 2020, couples an anti‐Trop‐2 antibody to the active metabolite of irinotecan (SN‐38). In the Phase III ASCENT trial, SG significantly improved outcomes in patients with triple‐negative breast cancer (TNBC), nearly doubling median PFS and markedly extending OS compared with physician's choice chemotherapy (TPC) [5, 6]. This study provided the first evidence that an ADC could outperform standard chemotherapy in heavily pretreated metastatic TNBC, establishing SG as the new standard of care following prior lines of therapy. Datopotamab deruxtecan (Dato‐DXd, Datroway) is a Trop‐2‐targeted ADC approved for the treatment of unresectable or metastatic hormone receptor (HR)‐positive, HER2‐negative breast cancer, with ongoing clinical trials investigating its application in other subtypes such as TNBC. In the Phase III TROPION‐Breast01 trial, Dato‐DXd significantly improved PFS compared with chemotherapy (∼37% risk reduction), although no statistically significant difference in OS was observed [7]. These results support the continued investigation of Dato‐DXd in combination regimens and across various breast cancer subtypes.

Growing clinical evidence indicates that tumors can develop both primary and acquired resistance to ADCs, ultimately limiting response durability. A review of phase III trials involving the four approved ADCs (T‐DM1, T‐DXd, SG, and Dato‐DXd) reveals a consistent pattern of strong initial responses followed by resistance. In HER2‐positive breast cancer, both T‐DM1 and the more potent T‐DXd demonstrated meaningful clinical benefit, although resistance eventually emerged. Similar trends were observed with SG in TNBC and Dato‐DXd in HR‐positive/HER2‐low breast cancer, where early improvements in PFS were later offset by tumor progression. These outcomes highlight the importance of understanding resistance mechanisms to improve the long‐term efficacy of ADC therapies. To tackle these challenges, early investigations primarily focused on tumor‐intrinsic mechanisms, such as the loss of the target antigen or increased expression of drug efflux pumps, which diminishes ADC effectiveness [8, 9]. However, resistance is now increasingly understood as a multifactorial process. The TME can serve as both a physical barrier to ADC penetration and a protective niche for cancer cells during treatment. Recent advances in spatial omics, single‐cell multi‐omics, and functional genomic technologies now enable systematic identification of resistance determinants and inform rational ADC selection strategies. At the same time, innovative linker designs and immune‐modulating approaches are emerging as key opportunities to overcome resistance by reshaping drug delivery and immune responses within the tumor microenvironment [10, 11]

Recent reviews, including that by Valle et al., have comprehensively summarized resistance mechanisms and future therapeutic perspectives of ADCs in breast cancer [8, 12, 13, 14]. Building on this foundation, the present review focuses on how resistance—particularly shaped by the tumor microenvironment—can be systematically interrogated using advanced methodologies and translated into rational guidance for ADC selection and therapeutic design. Accordingly, we outline established resistance pathways and extend the discussion to underappreciated contributors, including tumor microenvironmental influences, clonal evolution, and epigenetic and metabolic reprogramming. We highlight emerging experimental tools that not only deepen our understanding of resistance mechanisms but also serve as powerful platforms for preclinical and translational modeling to predict ADC responses and guide optimal ADC selection. We further explore how rational linker design can modulate immune activation and reshape the tumor microenvironment. Finally, we propose rational strategies to overcome resistance and outline future directions—emphasizing a holistic, ecosystem‐level approach to ADC efficacy. By addressing the multifaceted nature of resistance, we aim to unlock the full potential of ADCs for durable breast cancer treatment.

## 2 Resistance Mechanisms: An Eco‐system Perspective

Initial studies of ADC resistance have revealed multiple tumor cell–intrinsic mechanisms that undermine therapeutic efficacy. These mechanisms often align with the sequential steps required for ADC function: target antigen recognition and binding, internalization and trafficking to lysosomes, payload release, and engagement with the molecular target. Disruption at any of these stages can significantly reduce ADC potency. However, increasing evidence indicates that such tumor cell–intrinsic resistance mechanisms do not operate in isolation but are instead closely influenced by the TME. Antigen loss, lysosomal alterations, and drug efflux, therefore, represent interconnected processes shaped by microenvironmental pressures. Key TME features, such as hypoxia, extracellular acidity, and immune suppression, can modulate antigen expression, receptor internalization, and payload activation, thereby collectively limiting ADC efficacy.

One of the most direct mechanisms by which cancer cells can evade ADCs is by reducing or losing the target antigen, thereby impairing ADC binding and internalization. This phenomenon has been observed both clinically and in preclinical models. In HER2‐positive breast cancer, resistance to T‐DM1 is frequently associated with reduced HER2 expression or loss of HER2 gene amplification in residual tumor cells. Clinical reports have described tumors that progress from HER2‐positive to HER2‐low or HER2‐negative, reflecting selective pressure against HER2‐expressing cells. This report aligns with the established observation that low HER2 expression predicts poor response to T‐DM1 [15]. Similarly, in TNBC treated with SG, rare clones with Trop‐2 antigen alterations have been identified. Notably, a TROP2 T256R missense mutation was detected in a patient who developed resistance to SG, and this mutation impaired Trop‐2 antibody binding, effectively rendering the tumor resistant to Trop‐2‐targeted ADCs [16]. Such antigen escape may lead to cross‐resistance among ADCs targeting the same epitope, underscoring the importance of alternative targeting strategies in cases of relapse. Beyond genetic mutations or deletions, antigen expression can be downregulated through transcriptional or post‐transcriptional mechanisms. Prolonged exposure to T‐DM1 has been shown to suppress HER2 mRNA and protein expression in vitro, suggesting an adaptive feedback mechanism that diminishes target availability [17]. Epigenetic silencing mechanisms, such as promoter methylation, may also contribute to antigen loss. Furthermore, intratumoral heterogeneity enables the selective outgrowth of pre‐existing antigen‐low subclones during treatment, a Darwinian selection process in which tumor cells with reduced target expression gain a survival advantage under therapeutic pressure [18]. Heterogeneous HER2 expression across metastatic sites highlights the challenge of incomplete target coverage, allowing survival of tumor cell subsets that do not respond to HER2‐targeted therapy.

Recent studies have reinforced lysosomal dysfunction as a central mechanism of resistance to non‐cleavable ADCs, such as T‐DM1. In resistant cancer cells, internalized T‐DM1 often becomes sequestered in dysfunctional lysosomes with elevated pH and reduced protease activity, thereby impairing the proteolytic release of its cytotoxic payload. These alterations reduce cathepsin activity and attenuate production of the active DM1 catabolite, ultimately weakening microtubule inhibition and cytotoxic efficacy [19]. Pharmacological elevation of lysosomal pH, for example, by inhibiting V‐ATPase, can induce resistance even in otherwise sensitive cells, highlighting the critical role of an acidic lysosomal environment for effective ADC activation [20].

Beyond enzymatic activity, lysosomal transporters also play a key role. Notably, downregulation of the lysosomal membrane transporter SLC46A3 has been identified as a recurrent mechanism of resistance in T‐DM1‐resistant models [21, 22]. SLC46A3 is essential for exporting lysine‐MCC‐DM1, a key catabolite, from the lysosome to the cytosol. Its absence results in intracellular trapping of the inactive payload, whereas restoring SLC46A3 expression re‐sensitizes cells to T‐DM1. Recent mechanistic studies have further revealed that SLC46A3 acts as a proton‐coupled transporter, facilitating lysine‐DM1 efflux and providing a clear explanation for its role in mediating payload release [23]. Complementary genome‐wide CRISPR screens have identified genes involved in lysosomal acidification, protease maturation, and transporter function as key determinants of ADC sensitivity, suggesting that cancer cells may adopt a “dormant lysosome” phenotype, successfully internalizing the ADC but failing to activate it.

In parallel, defects in intracellular trafficking represent another axis of resistance. Rather than following canonical endo‐lysosomal degradation pathways, some resistant cells misroute internalized ADCs to recycling endosomes or multivesicular bodies, thereby bypassing lysosomes altogether. This diversion may involve either recycling ADCs to the plasma membrane or their sequestration within caveolin‐1‐positive vesicles, which reduces their exposure to acidic, proteolytic compartments and allows exocytosis of intact ADCs [24]. These trafficking alterations further prevent drug release at the cytotoxic site of action.

Following intracellular release and engagement of the cytotoxic payload with its target, resistance mechanisms often resemble those seen with conventional chemotherapeutics. At this stage, the key determinant of therapeutic efficacy is whether tumor cells can withstand the cytotoxic insult. Resistance to the payload itself can emerge through several well‐characterized pathways.

One of the most prevalent mechanisms involves the overexpression of ATP‐binding cassette (ABC) transporters that actively expel cytotoxic agents from the cytoplasm. ADC payloads, once liberated, behave similarly to highly potent chemotherapy drugs and are thus susceptible to efflux by multidrug resistance (MDR) pumps. Efflux transporters, such as P‐glycoprotein (encoded by ABCB1) and breast cancer resistance protein (encoded by ABCG2), can actively export a broad range of cytotoxic payloads from the cytosol, thereby lowering intracellular drug concentrations and diminishing the efficacy of ADCs [25]. In the context of T‐DM1 resistance, several studies have demonstrated that resistant cell lines frequently exhibit upregulated MDR1 expression, and inhibition of MDR1 can restore sensitivity, confirming efflux as a critical resistance mechanism. Acquired T‐DM1 resistance in vitro has often been associated with concurrent HER2 downregulation and MDR1 overexpression [21]. Similarly, resistance to SG has been linked to upregulation of ABCG2, a transporter that effluxes camptothecin analogs such as SN‐38 [26]. Interestingly, deruxtecan (DXd), the payload of T‐DXd, appears less susceptible to MDR1‐mediated efflux than maytansinoids, which may potentially explain its retained efficacy in some T‐DM1‐resistant tumors [27]. Nonetheless, efflux‐mediated resistance remains a formidable barrier, especially under sustained drug pressure.

Another mechanism of payload resistance involves mutations in the payload's molecular target that prevent effective binding. A clinically notable example is the emergence of a TOP1 E418K mutation in a tumor following treatment with SG, conferring resistance to SN‐38 and likely other topoisomerase I inhibitors [16]. Analogous mechanisms have been well‐documented in traditional chemotherapy; for example, mutations in β‐tubulin confer resistance to taxanes and maytansinoids, while mutations in topoisomerase II confer resistance to anthracyclines [28, 29]. Preclinical models of T‐DM1 resistance have similarly implicated tubulin mutations or altered β‐tubulin isoform expression in DM1 resistance. Target‐specific mutations typically induce cross‐resistance to all agents that share the same target but do not affect sensitivity to ADCs with payloads that use different mechanisms of action [30].

Beyond discrete mutations, cancer cells can activate adaptive survival pathways that mitigate the cytotoxic effects of payload‐induced damage. For DNA‐damaging payloads, upregulation of DNA repair mechanisms, particularly homologous recombination repair (HRR), has been implicated. In the context of SG, tumors proficient in HRR tend to have shorter response durations compared to those deficient in HRR [31]. Clinical correlative analyses from the IMMU‐132 trial indicated that high expression of DNA repair genes predicted reduced clinical benefit from SG, suggesting that both baseline and acquired DNA repair capacity can drive resistance [31]. Similarly, for microtubule‐inhibiting payloads, resistance can arise through evasion of apoptosis despite mitotic arrest, mediated by the overexpression of anti‐apoptotic BCL‐2 family proteins or the loss of pro‐apoptotic factors, such as BIM [32, 33]. Activation of survival pathways, including the PI3K/AKT signaling pathway, may further protect cells from payload‐induced stress and support resistance [34, 35].

While these resistance mechanisms are often classified as tumor‐intrinsic, accumulating evidence indicates that they are dynamically shaped and reinforced by tumor microenvironmental cues. In particular, antigen expression alone does not fully capture the complexity of ADC responsiveness, as efficient antibody–antigen internalization is equally critical for therapeutic activity. Under hypoxic conditions within the tumor microenvironment, phosphorylated caveolin‐1 redistributes to the plasma membrane, impairing trastuzumab internalization and reducing T‐DM1 cytotoxicity [36]. In parallel, hypoxia‐driven stabilization of hypoxia‐inducible factor‐1α (HIF‐1α) directly suppresses the expression of ATP6V1A, a key V‐ATPase subunit required to maintain lysosomal homeostasis, causing lysosomal dysfunction that could impair ADC payload activation [37].

**FIGURE 1:**
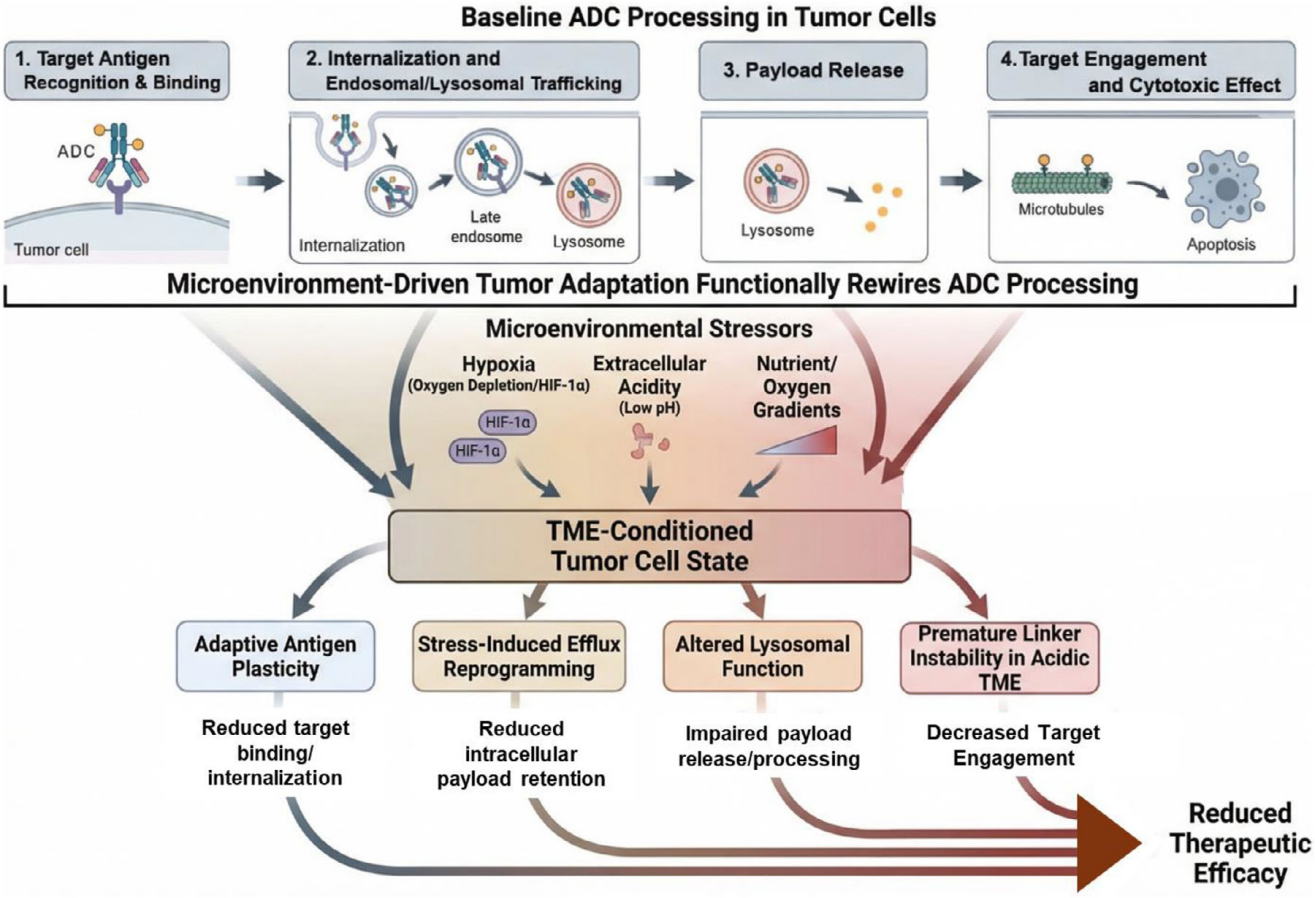
Tumor microenvironment–driven modulation of ADC processing and efficacy. The top panel depicts the canonical steps of ADC activity, including antigen binding, internalization, and endosomal–lysosomal trafficking, payload release, and cytotoxic target engagement. The lower panel illustrates how microenvironmental stressors such as hypoxia, extracellular acidity, and nutrient/oxygen gradients induce a TME‐conditioned tumor cell state that alters antigen expression, drug efflux, and lysosomal function. At the same time, acidic conditions may also compromise linker stability. These interconnected effects impair intracellular payload activity and reduce therapeutic efficacy, underscoring that ADC resistance arises from dynamic interactions between tumor‐intrinsic pathways and the tumor microenvironment rather than from independent mechanisms.

The modulation of tumor‐intrinsic determinants of ADC resistance by the tumor microenvironment is schematically illustrated in Figure 1.

### 2.1 Tumor Microenvironment as a Dynamic Modulator

The TME, composed of stromal cells (e.g., fibroblasts, endothelial cells), immune infiltrates, and the extracellular matrix (ECM), plays a critical role in modulating ADC distribution and activity within tumors. Various TME components have been implicated in limiting ADC efficacy or contributing to resistance mechanisms. Among them, tumor‐associated macrophages (TAMs), which frequently infiltrate breast tumors in high numbers, exert pro‐tumoral functions, including immunosuppression, promotion of invasion, and modulation of tissue structure. TAMs can influence ADC activity through multiple mechanisms. TAMs exhibit an M2‐like immunosuppressive phenotype, secreting cytokines and enzymes such as IL‐10, TGF‐β, and ARG1, which inhibit the function of cytotoxic CD8^+^ T cells and promote the recruitment and expansion of regulatory T cells (Tregs), thereby suppressing anti‐tumor immunity and ultimately reducing ADC efficacy [38, 39]. They also contribute to the maintenance of abnormal, leaky, and poorly organized vascular structures by producing pro‐angiogenic and matrix‐remodeling factors such as VEGF, MMP9, and PDGF, which further impair ADC distribution and limit drug penetration into the tumor core [40, 41]. Notably, TAMs express Fcγ receptors, enabling them to bind and internalize ADCs, effectively acting as a “sink” that reduces the availability of drugs to antigen‐expressing tumor cells. TAMs can also secrete cathepsins and other proteolytic enzymes that cleave ADC linkers prematurely within the TME, leading to off‐target payload release and diminished delivery to antigen‐positive tumor cells, thereby contributing to therapeutic resistance. In particular, high densities of CCR2^+^ TAMs have been linked to aggressive breast cancer phenotypes and immunosuppressive microenvironments. These macrophage subsets may not only sequester ADCs but also release survival‐promoting signals that help tumor cells resist exposure to sublethal payloads [42].

Cancer‐associated fibroblasts (CAFs), which are abundant in many breast tumors, particularly in certain subtypes such as TNBC and lobular carcinoma, play a central role in remodeling the TME. CAFs secrete ECM components, including collagen and fibronectin, leading to the formation of a dense, fibrotic stroma. This dense ECM poses a significant barrier to the penetration of large therapeutic molecules such as ADCs. Recent studies have demonstrated that excessive CAF activity leads to the formation of a fibrotic barrier, markedly limiting ADC uptake by tumor cells. In addition to serving as a physical barrier, CAFs are a major source of immunoregulatory cytokines, most notably TGF‐β. CAF‐derived TGF‐β sustains fibrotic ECM programs, limits intratumoral T‐cell infiltration, suppresses antigen presentation, and restrains the acquisition of TH1‐polarized effector phenotypes, collectively impairing productive anti‐tumor immunity [43]. These CAF‐driven, TGF‐β–dependent mechanisms have important implications for ADC therapy, as stromal fibrosis can restrict ADC penetration, while concomitant immune suppression limits immune‐mediated tumor clearance following ADC‐induced cytotoxicity or immunogenic cell death.

The TME is often enriched with immunosuppressive elements, including T regulatory cells (Tregs), myeloid‐derived suppressor cells (MDSCs), and inhibitory cytokines, such as TGF‐β, IL‐10, and IL‐6, which collectively dampen the activity of effector immune cells by inhibiting T cell proliferation, impairing cytotoxic function, and promoting immune cell exhaustion. Although such immunosuppressive mechanisms are primarily recognized for impairing the efficacy of immunotherapies, they can also indirectly influence the therapeutic outcomes of ADCs. For instance, if an ADC induces immunogenic cell death (ICD) and promotes the release of tumor‐associated antigens, a highly suppressive TME rich in anti‐inflammatory cytokines may prevent effective dendritic cell maturation, antigen presentation, and subsequent priming of tumor‐specific T cells, thereby limiting secondary tumor clearance. Notably, under certain conditions, ADC‐mediated ICD can reprogram an initially immunologically “cold” TME into a “hotter” and more inflamed microenvironment, thereby enhancing immune‐mediated tumor rejection [44]. This transition is often accompanied by the transient induction of pro‐inflammatory cytokines (e.g., type I interferons, IL‐12, and TNF‐α), which support antigen presentation and effector T cell recruitment; however, sustained immunosuppressive cytokine signaling may counteract this process and restore immune tolerance. The dynamic balance between these opposing forces —immunosuppression versus immune activation —may critically determine long‐term therapeutic outcomes. Specifically, when T cell activation is rapidly suppressed by cytokine‐mediated reinforcement of Treg and MDSC function, residual tumor cells may evade immune surveillance and eventually drive tumor recurrence.

Large tumors often exhibit regions of hypoxia (low oxygen tension) and extracellular acidosis (low pH), primarily due to aberrant and inefficient vasculature. Hypoxia contributes to tumor aggressiveness and therapy resistance by activating hypoxia‐inducible factor 1 (HIF‐1)‐regulated gene expression programs [45]. In hypoxic conditions, tumor cells may enter a quiescent state, reducing their susceptibility to cell‐cycle‐dependent cytotoxic agents. Simultaneously, hypoxia induces the expression of genes such as MDR1 and VEGF, which can alter drug transport and promote abnormal vascular permeability, potentially impairing ADC delivery and efficacy [46, 47]. Acidosis within the TME can further complicate ADC performance. A low extracellular pH may inactivate specific payloads or alter antibody binding affinity. In ADCs utilizing acid‐sensitive linkers, extracellular acidity could trigger premature cleavage and payload release outside target cells, thereby increasing off‐target toxicity and reducing intracellular delivery [48]. Additionally, both hypoxia and acidosis can promote ECM remodeling by activating TGF‐β and other profibrotic signaling pathways, reinforcing physical barriers to drug penetration [49].

In summary, the TME is not merely a passive backdrop in ADC therapy but an active participant that can significantly impede therapeutic efficacy. The TME plays a multifaceted role in modulating the overall effectiveness of ADCs, extending beyond physical delivery barriers. Components of the TME can influence antigen accessibility, alter linker or payload stability, activate cellular resistance mechanisms, and suppress immune‐mediated anti‐tumor responses. These factors collectively shape therapeutic outcomes and may explain heterogeneous responses observed in clinical settings.

### 2.2 Adaptive and Dynamic Resistance of Cancer Cells

Tumor cell populations are inherently dynamic and can evolve in response to therapeutic pressure. While ADC treatment may induce substantial tumor regression, the residual tumor cells, whether harboring pre‐existing resistance traits or undergoing adaptive changes, often repopulate the tumor, frequently in a more treatment‐refractory form. Two key biological processes underlie this phenomenon: clonal evolution, which involves the selection and expansion of resistant subclones, and phenotypic plasticity, the ability of cancer cells to alter their functional states without permanent genetic alterations, enabling reversible changes.

In large tumors, it is likely that rare subpopulations with inherent resistance traits, such as reduced target antigen expression or elevated efflux transporter levels (e.g., MDR1), pre‐exist before therapy. ADC treatment selectively eliminates sensitive clones, allowing the expansion of these minor populations through a process referred to as pre‐existing resistance. In contrast, cancer cells may also acquire resistance through adaptive mechanisms, such as activating survival pathways or undergoing phenotypic state transitions in response to sublethal drug exposure. For instance, prolonged in vitro exposure to T‐DM1 has been shown to downregulate HER2, suggesting a dynamic, therapy‐induced phenotypic shift [50]. Single‐cell transcriptomic analyses can help distinguish between these resistance types: a sharp post‐treatment enrichment of a rare pre‐existing trait implies selection, while the emergence of entirely new phenotypes suggests induction or plasticity‐driven switching. Phenotypic plasticity in cancer encompasses a broad spectrum of transitions, including epithelial‐to‐mesenchymal transition, during which tumor cells lose epithelial markers such as HER2 or Trop‐2 and acquire migratory and invasive properties. Under therapeutic pressure, particularly from ADCs, HER2‐positive tumors may transition to a HER2‐low or triple‐negative‐like phenotype [51, 52].

Furthermore, ADC therapy has been shown not only to exert cytotoxic effects but also to induce phenotypic adaptations that contribute to acquired resistance. Recent work demonstrated that treatment of HER2‐positive breast and gastric cancer models with a panel of HER2‐targeting ADCs—including trastuzumab emtansine, trastuzumab deruxtecan, XMT‐1522, and disitamab vedotin—leads to the formation of polyploid giant cancer cells (PGCCs) that exhibit characteristics of drug‐tolerant persister cells and survive prolonged ADC exposure. These PGCCs displayed reduced HER2 expression and attenuated HER2 amplification, consistent with treatment adaptation, and were able to sustain drug resistance in vitro and in vivo [53]. Importantly, transcriptomic and protein profiling revealed upregulation of nectin‐4 in ADC‐induced PGCCs relative to parental tumor cells, suggesting that this cell state expresses alternative surface antigens that could be therapeutically exploited. Supporting this, subsequent treatment with enfortumab vedotin, a nectin‐4–targeted ADC, effectively inhibited regrowth of PGCC‐driven xenograft tumors, indicating a potential vulnerability in ADC‐resistant disease. Together, these studies highlight a novel resistance axis in ADC therapy and identify PGCC‐associated markers, such as nectin‐4, as potential targets to overcome resistance and improve clinical outcomes.

Cancer cells can undergo heritable changes in gene expression without altering the underlying DNA sequence, a phenomenon known as epigenetic modifications, which serves as a mechanism for developing drug resistance. They can also rewire their metabolism to better cope with therapeutic stress. Epigenetics encompasses DNA methylation, histone modifications, and chromatin remodeling, all of which work together to regulate gene expression programs. Under chronic drug exposure, tumor cells may epigenetically silence specific genes or activate others to adapt to the environment. For example, in the context of ADC resistance, one plausible epigenetic mechanism is silencing the gene encoding the target antigen. If the promoter of HER2 (ERBB2) were to become hypermethylated, the cell would produce less HER2, effectively mimicking a genetic loss [54]. Another example involves the upregulation of drug resistance genes, such as MDR1, through chromatin changes that increase activity at the MDR1 locus [55, 56]. Acquired drug resistance in various cancers has been associated with promoter hypomethylation of efflux pump genes or hypermethylation of apoptotic genes. Epigenetic plasticity enables some tumor cells to enter transient, drug‐tolerant states, often through changes in histone modifications that suppress proliferation and promote stress adaptation. Notably, such epigenetically regulated states can sometimes be reversed with agents such as HDAC inhibitors or demethylating agents. However, the therapeutic benefit depends on the specific gene targets affected by these interventions [57]. For example, HDAC inhibitors have been shown to increase the expression of specific surface antigens. If a tumor reduces Trop‐2 expression, an epigenetic drug could potentially restore Trop‐2 expression, thereby restoring ADC binding and efficacy [58]. Similar approaches could be explored in breast cancer, where DNA methyltransferase (DNMT) inhibitors or HDAC inhibitors may increase HER2 or Trop‐2 levels in tumors with low baseline expression [59, 60]. Moreover, epigenetic therapies could potentially reduce tumor heterogeneity by promoting more uniform expression states across tumor cells.

**FIGURE 2:**
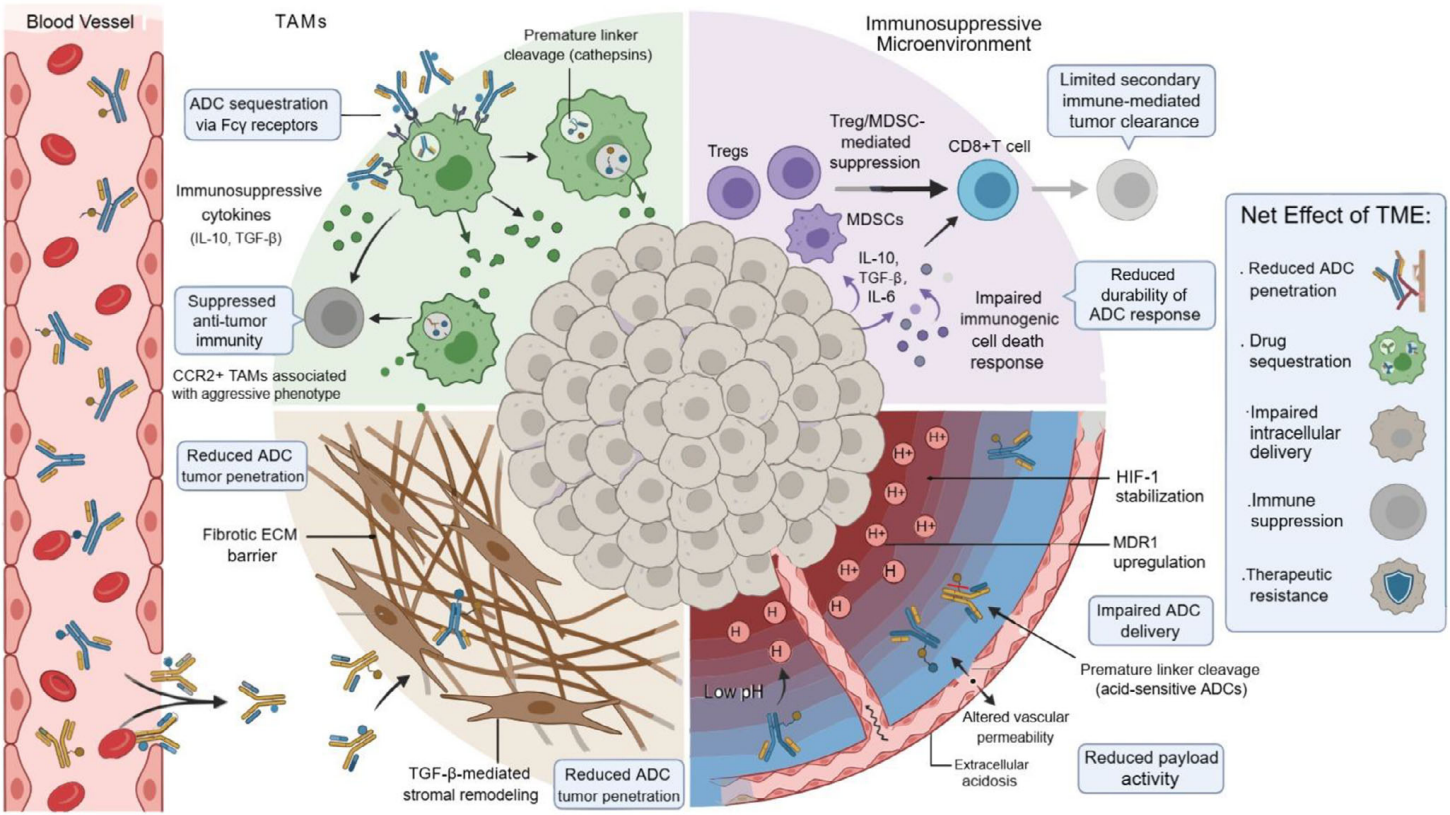
Tumor microenvironment–driven barriers to ADC efficacy. Components of the TME limit ADC activity through multiple mechanisms. TAMs sequester ADCs and promote premature linker cleavage, while immunosuppressive cells, such as Tregs and MDSCs inhibit CD8^+^ T‐cell–mediated tumor clearance. TGF‐β–driven stromal remodeling creates a dense ECM that restricts ADC penetration and tumor uptake. In parallel, hypoxia‐associated changes, including HIF‐1 stabilization, extracellular acidosis, and MDR1 upregulation, impair intracellular drug delivery and payload activity. Together, these processes reduce ADC penetration, enhance drug sequestration, and promote therapeutic resistance.

To highlight the underexplored yet potentially significant contributors to ADC resistance, we generated a schematic Figure 2 illustrating mechanisms, such as tumor microenvironment‐associated factors. These processes, though less well characterized than classical resistance pathways, may play a critical role in therapeutic failure and warrant further investigation.

## 3 Methodological Advances in Understanding ADC Resistance

Deciphering the complex interplay of factors that govern ADC response or resistance requires innovative research tools beyond traditional models. Conventional cell lines and bulk tumor analyses fall short of capturing tumors' spatial and cellular heterogeneity or the wide range of genes that influence drug response. Recent advances in cutting‐edge technologies enable high‐resolution analysis of tumors at both the spatial and single‐cell levels. These tools also enable systematic gene perturbation, allowing researchers to identify previously unrecognized mechanisms of drug resistance. Notably, spatial transcriptomics/proteomics, single‐cell omics, functional genomic screens, and organoid or co‐culture models are transforming our understanding of ADC mechanisms and resistance. These approaches provide previously unattainable insights, offering new avenues for improving ADC efficacy.

### 3.1 Advanced Tools for Dissecting Resistance: Spatial Omics

Recent advances in spatial omics technologies have transformed our ability to interrogate therapeutic resistance by preserving the spatial organization of tumors rather than dissociating them into single‐cell suspensions. Platforms such as 10x Genomics Visium, Slide‐seq, MERFISH, NanoString GeoMx, Imaging Mass Cytometry, and CODEX enable multiplexed transcriptomic and proteomic profiling within intact tissue architecture, allowing direct visualization of how tumor, stromal, and immune compartments interact in situ [61]. This spatial context is particularly critical for understanding resistance to ADCs, whose efficacy depends not only on target expression but also on tissue accessibility, microenvironmental barriers, and immune engagement.

Although spatial studies specifically addressing ADC resistance remain limited, emerging evidence demonstrates their ability to uncover clinically meaningful resistance programs. In HER2‐positive gastric tumors treated with trastuzumab and T‐DXd, spatial transcriptomics identified discrete intratumoral regions enriched for epithelial‐to‐mesenchymal transition signatures, activation of the endoplasmic reticulum‐associated degradation (ERAD) pathway, and diminished antigen‐presentation machinery [62]. These spatially confined programs suggest that resistance can emerge within localized niches that simultaneously buffer cytotoxic stress and evade immune surveillance.

Spatial profiling has further revealed that the distribution of ADC‐relevant antigens is highly heterogeneous. In triple‐negative breast cancer (TNBC), nine spatial tumor archetypes have been described based on differential expression of Trop‐2 and Nectin‐4—both clinically actionable ADC targets—alongside distinct stromal structures and immune‐exclusion patterns associated with patient outcomes [63]. Such findings highlight how antigen accessibility, stromal density, and immune contexture vary across tumor regions, collectively shaping ADC penetration, bystander activity, and immune‐mediated tumor clearance.

Additional insights come from spatial analyses of the HER2‐targeting ADC SHR‐A1811. Tumors enriched in tumor‐infiltrating lymphocytes, particularly cytotoxic T cells, were more likely to respond in HR‐negative patients, whereas HR‐positive tumors containing densely clustered HER2‐high regions showed poorer outcomes [64]. These observations indicate that antigen abundance alone is insufficient to predict ADC response in the absence of a permissive immune microenvironment, emphasizing the need to evaluate spatial immune context alongside target expression.

Beyond static resistance features, spatial omics also captures dynamic therapeutic remodeling. In TNBC, integration of spatial proteomics with single‐cell transcriptomics identified distinct immune response trajectories following anti‐PD‐1 therapy and radiotherapy, including tumors that initially failed checkpoint blockade but became responsive after treatment‐induced spatial reorganization enhanced immunogenicity [65]. Such findings underscore how therapeutic sensitivity can depend on the spatial evolution of the tumor microenvironment rather than baseline molecular features alone.

As spatial omics becomes increasingly integrated into translational research, longitudinal and multimodal spatial analyses will be essential for distinguishing pre‐existing resistant niches from therapy‐induced spatial reprogramming. Defining these spatial determinants of resistance is likely to guide biomarker‐driven, spatially informed combination strategies designed to overcome microenvironment‐mediated therapeutic failure.

### 3.2 Advanced Tools for Dissecting Resistance: Single‐cell Multi‐Omics

Single‐cell omics technologies—including scRNA‐seq, CITE‐seq, and scATAC‐seq—provide powerful approaches for resolving the cellular heterogeneity that underlies ADC resistance. Recent advances in multi‐omic integration now enable high‐resolution profiling of tumor ecosystems and the TME, allowing mechanistic dissection of resistance programs across molecular layers. By integrating transcriptomic, epigenomic, and proteomic information at single‐cell resolution, these methods illuminate cellular states, lineage relationships, and cell–cell interactions that collectively shape therapeutic response. As highlighted by recent reviews [66, 67], single‐cell technologies have become indispensable for understanding how lineage plasticity, state transitions, and tumor–stroma crosstalk drive cancer progression and treatment failure.

Single‐cell RNA sequencing (scRNA‐seq) enables unbiased transcriptional profiling of individual cells, thereby identifying rare subpopulations that are often masked in bulk analyses. In the context of ADC resistance, post‐treatment scRNA‐seq can reveal small tumor cell clusters harboring resistance‐associated transcriptional programs, such as increased expression of drug efflux transporters or reduced target‐antigen levels. In ER‐positive tumors, tamoxifen‐resistant subpopulations with distinct transcriptional signatures have been identified, and enrichment of these signatures in bulk datasets correlates with poor prognosis [68]. Serial scRNA‐seq analyses have further tracked the evolution of resistance during endocrine therapy combined with CDK4/6 inhibition, where non‐responders exhibited a shift from canonical ER signaling toward alternative survival pathways involving JNK and ERBB4 [69]. Similarly, in TNBC, chemoresistance arises through both pre‐existing resistant clones and therapy‐induced transcriptional reprogramming, highlighting the combined roles of clonal selection and cellular plasticity [70]. Sequential analyses of stage IV breast cancer samples have also revealed metastasis‐primed populations characterized by convergent chromosomal alterations and activation of epithelial‐to‐mesenchymal transition programs following chemotherapy [71]. Beyond resistance itself, scRNA‐seq has uncovered immune‐evasion and metastatic phenotypes, including stem‐like CD44^+^/ALDH2^+^/ALDH6A1^+^ subpopulations and NECTIN–TIGIT ligand–receptor interactions that may facilitate immune escape [72]. Together, these findings demonstrate how transcriptional heterogeneity and dynamic cell‐state transitions contribute to therapeutic failure while informing rational ADC development and patient stratification.

CITE‐seq extends these capabilities by enabling simultaneous quantification of cell‐surface proteins and mRNA within the same single cells. Introduced by Stoeckius et al. in 2017, this approach uses oligonucleotide‐labeled antibodies to barcode epitopes that are sequenced alongside the cellular transcriptome [73]. By integrating phenotypic and transcriptional data, CITE‐seq overcomes key limitations of flow cytometry and conventional scRNA‐seq, enabling high‐dimensional profiling of tumor ecosystems without reliance on fluorescence‐based detection. This multimodal resolution is particularly relevant to ADC research because it allows direct measurement of surface antigen abundance while simultaneously interrogating intracellular survival signaling. As a result, CITE‐seq can distinguish whether resistant cells evade ADC‐mediated killing through antigen downregulation or through persistence of intrinsic survival pathways despite maintained antigen expression.

Clinically, CITE‐seq has been applied to breast cancer biopsies collected during anti–PD‐1 therapy, where integrated transcriptomic and proteomic profiling mapped immune states associated with response and nonresponse and revealed dynamic remodeling of the TME [74]. In TNBC models, CITE‐seq–based trajectory analyses following chemotherapy priming identified distinct myeloid programs linked to immune checkpoint blockade resistance and highlighted actionable regulators such as STAT1 [75]. These studies illustrate how multimodal single‐cell profiling can uncover resistance‐associated cellular states and microenvironmental programs that are not readily resolved by transcriptomic analysis alone. Longitudinal CITE‐seq analyses further enable pre‐ and post‐treatment comparisons to determine whether resistance arises from antigen loss, clonal expansion, or adaptive cell‐state transitions, thereby improving the detection of rare but clinically meaningful populations, such as minimal residual disease clones.

Single‐cell Assay for Transposase‐Accessible Chromatin using Sequencing (scATAC‐seq) provides complementary insight into epigenetic mechanisms of resistance by mapping chromatin accessibility at single‐cell resolution. Changes in chromatin structure—such as increased accessibility at the MDR1 promoter or reduced accessibility at target‐antigen loci—can underlie drug tolerance and therapeutic escape [76, 77]. Although scATAC‐seq studies specifically focused on ADC resistance remain limited, work in related therapeutic contexts provides important mechanistic parallels. In doxorubicin‐resistant MCF7 cells, ATAC‐seq identified more than 18 000 differentially accessible regions enriched for AP‐1 and FOX transcription factor motifs, implicating reprogramming of MAPK and PI3K–AKT signaling pathways [78]. In models of trastuzumab resistance, chromatin profiling revealed increased accessibility within the PPP1R1B locus, associated with upregulation of the tDarpp isoform—a truncated variant implicated in survival signaling and therapeutic resistance. These accessible regions were enriched for FOXJ3 and SOX2 binding motifs, transcription factors linked to stemness and cellular plasticity [79]. Extending beyond cell lines, integrated single‐cell ATAC‐seq and RNA‐seq analyses of matched primary and recurrent tumors have identified distinct cancer cell states emerging during disease progression and defined core gene signatures associated with endocrine resistance [80]. Collectively, these findings highlight the dynamic epigenomic evolution of tumor cells under therapeutic pressure and establish a framework for applying single‐cell chromatin profiling to elucidate mechanisms of ADC resistance.

### 3.3 Advanced Tools for Dissecting Resistance: Functional Screens

Functional genomic screening using CRISPR/Cas9 technologies, including genome‐wide knockout and CRISPR interference (CRISPRi) libraries, has become an essential strategy for systematically identifying genetic determinants of therapeutic response. In the context of ADCs, these unbiased approaches enable direct interrogation of the cellular machinery required for ADC processing and cytotoxic activity, thereby revealing resistance mechanisms that are often missed by candidate‐based studies. For example, regulators of endolysosomal trafficking, such as SLC46A3, were identified through CRISPR screening as unexpected mediators of ADC resistance [81]. While historically performed in vitro, these platforms are increasingly applied in vivo to capture resistance pathways shaped by physiologically relevant tumor microenvironments [82].

In TNBC, CRISPR screening of cells treated with SG demonstrated that loss of genes involved in lysosomal trafficking and antigen presentation impaired ADC cytotoxicity. In contrast, disruption of DNA damage repair genes such as PARP1 enhanced sensitivity. Notably, co‐targeting PARP1 significantly improved therapeutic efficacy when combined with the ADC [83]. Such findings illustrate how functional perturbation screens can distinguish genes required for ADC activity from those that buffer or counteract payload‐induced damage.

Mechanistically, CRISPR‐based screens link genotype to ADC response by selecting for gene perturbations that alter survival following treatment. Cells are then treated with ADCs, and gene knockouts that are enriched in the surviving population indicate genes essential for ADC activity. Enrichment of specific knockouts in surviving populations identifies processes essential for ADC efficacy, whereas depletion of other perturbations reveals vulnerabilities that can be therapeutically exploited. For example, deletion of a DNA repair gene can enhance the effect of a DNA‐damaging payload, supporting synergy with PARP inhibitors.

Since ADCs require multiple cellular processes such as antigen recognition, internalization, intracellular trafficking, release of the cytotoxic payload, and execution of cell death, CRISPR‐based functional screens uniquely enable systematic mapping of resistance nodes across this entire pathway. These approaches also help uncover synergistic drug‐gene combinations, supporting the rational design of combination strategies to overcome resistance and enhance clinical efficacy.

### 3.4 Advanced Tools for Dissecting Resistance: Organoids/Co‐Cultures

Patient‐derived organoids (PDOs), 3D cultures derived from patient tumors, have emerged as functionally relevant preclinical models for studying therapeutic response that preserve the histological architecture, genetic landscape, and intratumoral heterogeneity of the original tumors [84, 85, 86]. Compared with conventional 2D cell lines, organoids more accurately recapitulate tissue‐level features that directly influence ADC performance, including drug penetration barriers, cell‐cell interactions, and spatial antigen distribution. When combined with co‐culture systems that include stromal or immune components, organoids offer a powerful platform for studying ADC efficacy within a controlled yet physiologically contextualized tumor microenvironment [87].

In breast cancer research, organoid‐ or spheroid‐based 3D cultures have been used to investigate both ADC efficacy and resistance mechanisms. For example, HER2‐positive 3D cultures exhibit distinct responses to T‐DM1 compared with conventional 2D monolayers, including altered ADC uptake and increased heterogeneity in HER2 expression, underscoring the importance of 3D tumor organization in modulating ADC activity [88]. Similarly, Overmeer et al. established a panel of PDOs to evaluate HER2‐ and TROP2‐targeting ADCs and demonstrated that antigen expression levels within PDOs strongly predicted drug sensitivity [89]. The trafficking regulator SNX10 was further identified as a modulator of trastuzumab‐based ADC response in HER2‐positive PDO models; its loss impaired HER2 recycling, reduced surface antigen availability, and, consequently, diminished ADC binding, thereby promoting therapeutic resistance. Importantly, PDO systems preserved patient‐specific HER2 expression patterns, enabling direct functional validation of patient‐linked ADC vulnerabilities [90]. PDOs generated from matched pre‐ and post‐treatment biopsies also allow mechanistic dissection of acquired resistance while maintaining patient‐specific tumor context ex vivo [91].

In addition, emerging organoid‐based co‐culture platforms incorporating autologous immune cells, such as lymphocytes or macrophages, offer a promising framework for investigating immune‐related aspects of ADC activity, including immune cell recruitment and bystander effects mediated by membrane‐permeable payloads [87]. Although still in early stages of application to ADC research, such systems could provide valuable opportunities to interrogate how immune context and tumor architecture jointly shape ADC responses within heterogeneous tumor architectures.

Organoid models derived from normal tissues also represent a potential strategy for evaluating off‐target toxicity and safety liabilities during early‐stage ADC development. Furthermore, expanding patient‐derived organoid biobanks may enable scalable, high‐throughput screening of ADC candidates across genetically and phenotypically diverse tumor backgrounds, bridging mechanistic discovery with translational biomarker development and patient stratification.

As outlined in Table 1, emerging technologies provide complementary strategies to interrogate ADC resistance across multiple biological dimensions.

**TABLE 1:** Methodological Categories to Tackle ADC Resistance. These complementary methodologies enable multidimensional interrogation of ADC resistance across spatial, cellular, genetic, and microenvironmental levels, bridging mechanistic discovery with translational biomarker development.

Methodological category	Representative technologies	ADC resistance question	Mechanistic insight	Translational impact
Spatially Resolved Profiling	Spatial transcriptomics/proteomics (Visium, GeoMx, MERFISH, IMC, CODEX)	How does tumor architecture influence ADC delivery and activity?	Reveals regional antigen heterogeneity, stromal barriers, and immune exclusion affecting ADC penetration and efficacy.	Enables the development of spatial biomarkers and microenvironment‐informed combination strategies.
Single‐Cell Multi‐Omic Dissection	scRNA‐seq, CITE‐seq, scATAC‐seq	Which cellular subpopulations drive resistance?	Identifies rare resistant clones, lineage plasticity, antigen modulation, and adaptive survival programs masked in bulk analyses.	Supports patient stratification and the detection of minimal residual disease.
Functional Genomic Interrogation	CRISPR/Cas9 knockout, CRISPRi‐based screens	Which genes are required for ADC processing and cytotoxicity?	Maps pathways regulating antigen internalization, lysosomal trafficking, payload response, and DNA damage sensitivity.	Identifies actionable resistance nodes and rational drug combinations.
Physiologically Relevant Tumor Modeling	Patient‐derived organoids (PDOs), immune/stromal co‐cultures	How do tumor context and microenvironment shape ADC response?	Recapitulates the 3D structure, antigen distribution, and immune interactions that influence ADC pharmacobiology.	Provides functional validation of biomarkers and predictive preclinical testing platforms.

## 4 Strategies to Overcome Resistance

### 4.1 Dual‐Payload ADCs

One strategy to prevent or overcome payload‐specific resistance is to equip a single ADC with two distinct cytotoxic payloads. This concept is analogous to combination chemotherapy. The need for cancer cells to simultaneously resist two distinct mechanisms significantly reduces the likelihood of effective resistance. Dual‐payload ADCs have been experimentally investigated by conjugating two types of cytotoxins to the same antibody or by using hybrid linkers that carry bifunctional drugs. For example, a dual‐drug HER2 ADC that delivers both a DNA‐damaging pyrrolobenzodiazepine (PBD) and the tubulin inhibitor Monomethyl Auristatin E MMAE demonstrated superior efficacy in heterogeneous tumor models, including a strong bystander effect due to MMAE's membrane permeability [92]. In a landmark preclinical study, a HER2‐targeted ADC conjugated to both MMAE and MMAF via orthogonal click chemistry has been developed and shown to induce significantly greater tumor regression than either single‐payload ADC alone or the combination of the two single ADCs administered separately [93]. In another study, a trastuzumab‐based dual ADC carrying both MMAE and DM1 has been designed and shown to exhibit synergistic cytotoxicity in HER2‐positive breast cancer cell lines, even at subnanomolar concentrations [94]. These findings highlight the therapeutic advantage of simultaneous delivery versus co‐administration of separate agents. Beyond cytotoxins, dual‐payload ADCs are being explored to incorporate immune stimulators. A CD276‐targeted dual ADC that delivers both a microtubule inhibitor and a TLR7/8 agonist has been shown to induce direct tumor cell death and stimulate local immune activation in TNBC models, resulting in complete tumor regression in mice, an effect not achievable with the cytotoxic payload alone [95]. These “chemo‐immune” ADCs may offer novel ways to convert immunologically “cold” tumors into “hot” tumors. Comparative studies consistently show that dual‐payload ADCs outperform single‐payload ADCs in both efficacy and resistance circumvention. The co‐delivery mechanism ensures that each targeted cell receives both agents simultaneously, enabling synergistic effects and reducing the likelihood of survival via single‐pathway escape. While dual‐payload ADCs remain largely preclinical, they represent a promising strategy, particularly when combining a drug susceptible to efflux pumps with one that is not, or a lysosome‐activated drug with one that acts independently of lysosomal processing.

### 4.2 Bispecific and Biparatopic ADCs

Bispecific and biparatopic ADCs are emerging as promising strategies to overcome resistance and heterogeneity in breast cancer. These constructs are designed to either target two different antigens (bispecific) or two epitopes on the same antigen (biparatopic), offering enhanced tumor selectivity, payload delivery, and anti‐tumor activity compared to conventional monospecific ADCs.

Several bispecific and biparatopic ADCs have shown efficacy in preclinical and early‐phase clinical trials. A notable biparatopic ADC, ZW49 (zanidatamab zovodotin), targets two non‐overlapping HER2 epitopes and is conjugated to an auristatin payload. Preclinical data demonstrated potent cytotoxicity and internalization, even in HER2‐low models. In an early‐phase trial, ZW49 showed a confirmed ORR of approximately 28% in heavily pretreated HER2‐positive breast cancer patients, with manageable toxicity [96]. Another agent, MEDI4276, a biparatopic HER2‐targeted ADC with a tubulysin payload, showed strong preclinical efficacy but was limited in clinical application due to hepatotoxicity at higher doses [97].

In contrast, BL‐B01D1, a bispecific ADC targeting EGFR and HER3 with a topoisomerase I payload, demonstrated approximately 60% response rates in a Phase I trial involving solid tumors, including breast cancer, and showed durable responses in some cases of resistance [98]. Mechanistically, these dual‐targeting ADCs offer several advantages. First, biparatopic binding enhances receptor clustering and internalization, leading to more efficient intracellular delivery of the cytotoxic payload. This is especially valuable in HER2‐low or heterogeneous tumors, where traditional HER2‐targeted ADCs may be insufficient. Second, by recognizing multiple targets, bispecific ADCs can broaden tumor cell coverage and reduce the likelihood of antigen escape. This is critical in triple‐negative or treatment‐resistant breast cancers, which often exhibit intratumoral heterogeneity. Dual‐target ADCs also offer the potential to overcome resistance mechanisms. For example, HER2 truncation variants (e.g., p95HER2) may retain one epitope while losing another; biparatopic ADCs can still bind and exert effects. Moreover, co‐targeting EGFR and HER3 not only enhances cell binding but may also inhibit compensatory signaling pathways, reducing survival advantages conferred by resistance mutations.

In summary, bispecific and biparatopic ADCs are evolving as powerful next‐generation therapeutics in breast cancer. By addressing key limitations of traditional ADCs, including antigen heterogeneity and resistance, they hold promise for improving outcomes in difficult‐to‐treat cancer subtypes.

### 4.3 TME Modulators

TME plays a pivotal role in limiting the therapeutic efficacy of ADCs in breast cancer. ECM deposition by CAFs, Fcγ receptor–mediated sequestration by TAMs, immunosuppressive cytokines such as TGF‐β and IL‐10, and aberrant tumor vasculature collectively create physical and immunologic barriers that impair ADC penetration and function. These factors reduce intratumoral drug exposure and dampen immune activation following ADC‐induced tumor cell death, ultimately promoting therapeutic resistance.

To overcome these constraints, therapeutic strategies that remodel the TME rather than directly targeting tumor cells have emerged as a promising approach to enhance ADC activity.

One major strategy targets CAF‐driven stromal fibrosis, which acts as a physical barrier to ADC delivery. Pharmacologic inhibition of focal adhesion kinase (FAK), a key regulator of CAF activation and ECM production, has been shown to disrupt collagen deposition, lower interstitial pressure, and improve intratumoral ADC distribution [99]. The FAK inhibitor IN10018 effectively dismantled fibrotic barriers and enhanced tumor uptake of HER2‐ and TROP2‐directed ADCs, resulting in deeper tumor regressions, including complete responses in preclinical models of ADC‐resistant disease [99]. In parallel, direct stromal‐targeting approaches have been explored using fibroblast activation protein (FAP)–directed ADCs such as OMTX705, which selectively deplete CAF populations and promote CD8^+^ T‐cell infiltration, thereby augmenting antitumor immunity [100, 101].

Given the central role of TGF‐β in maintaining CAF activation and fibrotic ECM programs, TGF‐β signaling blockade represents another complementary strategy. Preclinical studies have demonstrated that pharmacologic inhibition of TGF‐β reprograms the TME toward a more immune‐permissive state, facilitating T‐cell infiltration and enhancing responses to immune‐based therapies [102, 103, 104, 105]. These findings support the concept that targeting CAF‐derived TGF‐β signaling can enhance the durability of ADC responses by coordinating stromal and immune remodeling.

In addition to fibroblast‐mediated barriers, TAMs represent a critical cellular mediator of ADC resistance. TAMs can sequester Fc‐containing therapeutics and sustain an immunosuppressive milieu, thereby limiting effective drug delivery and immune engagement. Targeting macrophage survival and differentiation pathways, particularly by inhibiting the CSF1/CSF1R axis, has been shown to reduce immunosuppressive TAM populations and shift macrophage polarization toward a more pro‐inflammatory phenotype [106, 107]. Zhu et al. demonstrated that CSF1R blockade reprogrammed tumor‐infiltrating macrophages, resulting in enhanced effector T‐cell infiltration and improved antitumor immune responses [108]. Similarly, Ries et al. reported that CSF1R inhibition depleted suppressive TAMs and remodeled the TME, leading to significant tumor growth inhibition [109]. Collectively, these studies suggest that TAM‐directed therapies may enhance ADC efficacy by reducing drug sequestration, alleviating immune suppression, and improving functional drug penetration.

Beyond stromal and myeloid targeting, broader physiologic normalization strategies are also under investigation. Therapeutic interventions aimed at modulating tumor acidity or hypoxia, including systemic buffering agents or oxygenation‐enhancing approaches, are being evaluated to determine whether normalization of pH and oxygen gradients can improve ADC delivery and activity [110, 111].

Together, these approaches highlight the potential of TME‐modulating strategies to circumvent microenvironment‐driven resistance mechanisms and enhance the therapeutic index of ADCs by coordinating the remodeling of stromal, immune, and physiologic tumor barriers.

### 4.4 Epigenetic Reprogramming Agents

One of the most promising emerging strategies to overcome resistance to ADCs involves combining them with epigenetic or metabolic modulators to enhance therapeutic efficacy. These agents can modulate tumor cell plasticity, reprogram gene expression, or alter metabolic states, thereby resensitizing resistant cancer cells to ADC‐mediated cytotoxicity. For example, histone deacetylase (HDAC) inhibitors increase histone acetylation, leading to chromatin relaxation and reactivation of previously silenced target genes, including those encoding ADC target antigens or components of pro‐apoptotic pathways. This epigenetic reprogramming can partially reverse resistance mechanisms. Reflecting this potential, the first ADCs engineered with HDAC inhibitors as payloads showed effective epigenetic modulation and potent anti‐tumor activity with minimal toxicity [112].

Similarly, DNA methyltransferase inhibitors (DNMTis), such as azacitidine, help preserve target gene expression by preventing promoter hypermethylation and maintaining an open chromatin state, which may help counter resistance to targeted therapies [113]. In addition, lysine‐specific demethylase 1 (LSD1) inhibitors prevent the demethylation of histone H3K4me1/2, maintaining transcriptionally active chromatin states and reprogramming the transcriptome. LSD1 inhibition has been shown to reduce cancer stemness and epithelial‐mesenchymal transition, promoting a more differentiated and less invasive phenotype and ultimately enhancing ADC sensitivity. Preclinical models have reported that LSD1 inhibitors disrupt interactions between LSD1 and transcriptional repressors such as SNAIL1, reducing tumor invasiveness and heterogeneity, and contributing to the reversal of ADC resistance [114].

Collectively, these examples underscore the potential of combining epigenetic agents with ADCs to reprogram resistant cancer cells and abrogate key resistance mechanisms, with encouraging evidence emerging from preclinical and early clinical studies.

### 4.5 Radiotherapy‐ and Immunotherapy‐Based Strategies

Strategies that enhance tumor immunogenicity and promote adaptive immune activation, therefore, represent a rational approach to restoring ADC sensitivity. In this context, combining ADCs with radiotherapy and immunotherapy has emerged as a promising avenue to convert cytotoxic tumor cell death into durable antitumor immune responses.

Certain ADCs, particularly those with cleavable linkers and membrane‐permeable payloads, can induce a bystander effect, extending cytotoxicity to neighboring antigen‐negative tumor cells and amplifying local tumor damage. Beyond direct cell killing, multiple ADC payload classes—including maytansine–, kinesin spindle protein (KSP) inhibitor–, and topoisomerase I inhibitor–based ADCs—have been shown to trigger ICD, characterized by calreticulin exposure, ATP and HMGB1 release, and activation of innate immune signaling pathways such as cGAS–STING. These processes promote dendritic cell maturation, antigen presentation, and the priming of tumor‐specific cytotoxic T‐cell responses [115, 116, 117]. When combined with radiotherapy, these immunogenic effects are further amplified. The combined effect of ADC‐ and radiotherapy‐induced tumor damage promotes tumor antigen release and immune priming by activating innate immune pathways such as cGAS–STING and TLR signaling, leading to dendritic cell maturation and enhanced tumor‐specific CD8^+^ T‐cell responses [118]. This immunogenic remodeling of the tumor microenvironment provides a strong mechanistic rationale for integrating immune checkpoint blockade into ADC‐based treatment strategies. Notably, ADC‐mediated tumor radiosensitization allows spatially precise chemo‐radio‐immunotherapy, enhancing radiotherapy‐induced immunogenicity and improving responses to immune checkpoint blockade in preclinical models [119].

Building on this mechanistic foundation, clinical trials have evaluated the efficacy of ADC–immune checkpoint inhibitor (ICI) combinations in HER2‐positive metastatic breast cancer. The phase II KATE2 trial assessed T‐DM1 with or without atezolizumab and showed no significant PFS benefit in the overall population; however, PD‐L1–positive patients derived meaningful improvements in PFS and ORR, supporting a biomarker‐guided approach [120]. These findings prompted the ongoing KATE3 phase III trial, which is restricted to PD‐L1–positive patients. A Phase Ib study combining T‐DM1 and pembrolizumab demonstrated manageable safety and modest clinical activity, although pulmonary toxicity remains a concern [121]. Beyond T‐DM1, T‐DXd is being explored in combination with ICIs in the DESTINY‐Breast07 trial. Early results suggest robust antitumor activity that appears less dependent on PD‐L1 status, potentially reflecting broader immunogenic effects driven by its potent cytotoxic payload and bystander activity [122]. Similarly, in the ASCENT‐04 phase III trial, the combination of sacituzumab govitecan (SG) with pembrolizumab significantly improved PFS compared with chemo‐immunotherapy in PD‐L1–positive TNBC, supporting ADC–ICIs as a promising first‐line strategy in this setting [123]. Collectively, these studies highlight distinct ADC‐ICI combination paradigms, in which the optimal therapeutic strategy may depend on ADC mechanism of action, payload properties, and the tumor immune microenvironment, rather than a one‐size‐fits‐all approach.

**FIGURE 3:**
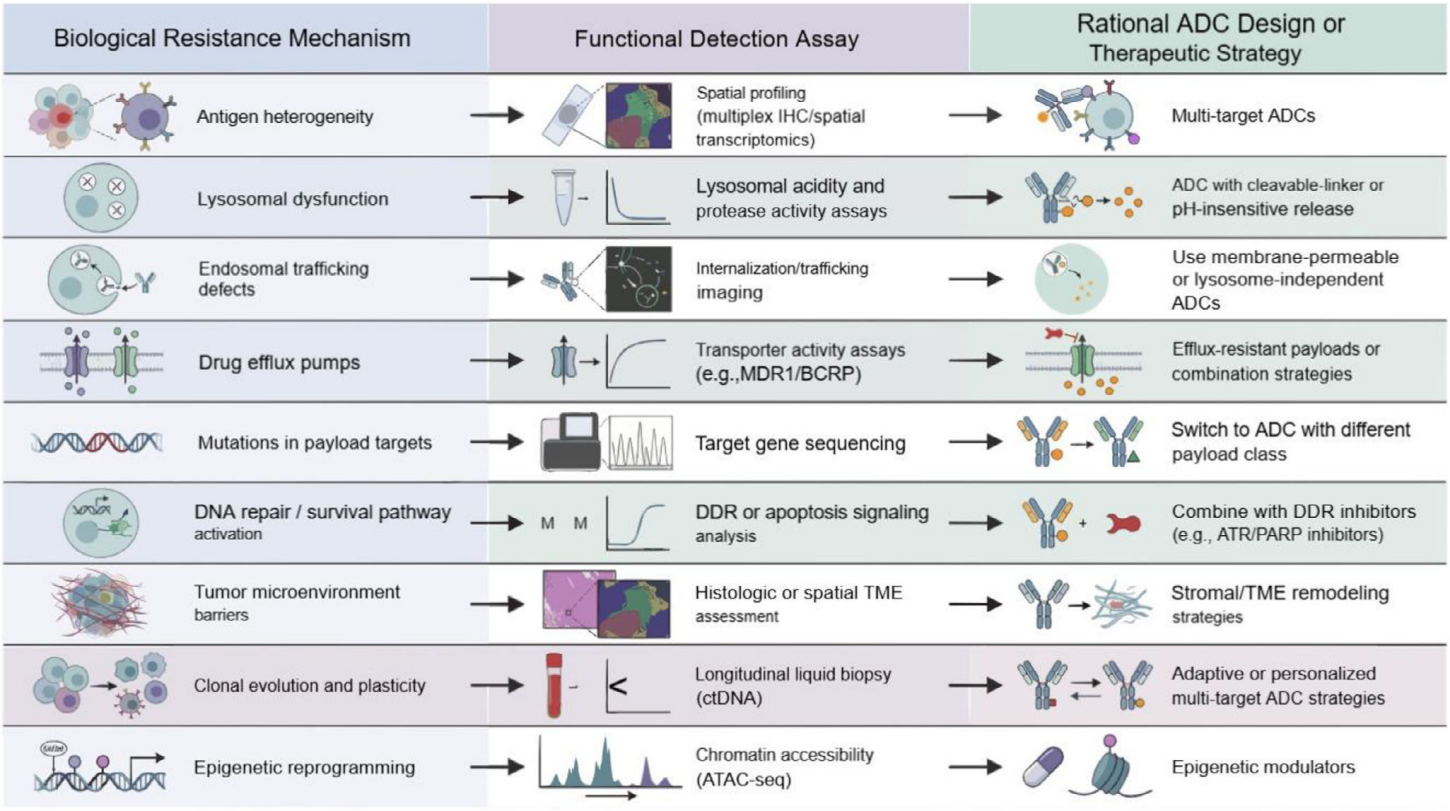
Mechanism‐informed detection and therapeutic strategies to overcome ADC resistance. Key biological mechanisms underlying resistance to ADCs can be systematically linked to functional assays and rational therapeutic interventions. Antigen heterogeneity, lysosomal dysfunction, endosomal trafficking defects, drug efflux activity, mutations in payload targets, and activation of DNA damage repair or survival pathways can be evaluated using spatial profiling, lysosomal and trafficking assays, transporter activity measurements, genomic sequencing, and signaling analyses. In parallel, tumor microenvironment barriers, clonal evolution, and epigenetic reprogramming can be assessed through histologic evaluation, longitudinal liquid biopsy, and chromatin accessibility profiling. These diagnostic insights inform mechanism‐based strategies, including multi‐target ADCs, linker or payload optimization, efflux‐resistant designs, combination therapies (e.g., DDR inhibitors), stromal remodeling, adaptive targeting approaches, and epigenetic modulation to enhance ADC efficacy.

As precision oncology advances, aligning resistance mechanisms with tailored diagnostic and therapeutic solutions becomes essential. Table 2 and Figure 3 outline a roadmap that connects mechanistic insights with actionable strategies to guide the development of next‐generation ADCs.

**TABLE 2:** Methodological Framework for Dissecting Mechanisms of ADC Resistance and Translational Opportunities. This table outlines key methodological approaches used to study resistance to ADCs, linking representative technologies to the biological questions addressed, the mechanistic insights gained, and their translational implications. Integrated spatial, single‐cell, functional genomic, and physiologically relevant modeling strategies collectively enable identification of resistance mechanisms, biomarker development, and rational design of next‐generation or combination therapies.

Resistance mechanism	Detection strategy	Treatment strategy
Antigen heterogeneity	Spatial profiling techniques (e.g., multiplex immunohistochemistry or spatial transcriptomics) can reveal intratumoral variation in antigen expression. Single‐cell RNA‐seq to identify antigen‐low or antigen‐negative subclones.	Use multi‐targeted approaches, such as bispecific or trispecific ADCs, to broaden coverage of heterogeneous tumor cells. Sequential or combinatorial ADCs targeting primary and compensatory antigens.
Lysosomal dysfunction	Measure lysosomal acidity and protease activity. Measure lysosomal acidification using LysoSensor probes and assess protease activity (e.g., cathepsin B/L).	Deploy ADCs with cleavable linkers or pH‐insensitive release mechanisms. Use payloads less dependent on lysosomal processing Include payloads capable of cytosolic release independent of lysosomal proteolysis.
Endosomal rerouting and intracellular trafficking dysregulation	Analyze internalization pathways via imaging or co‐localization studies.	Engineer linkers or payload chemistries that promote membrane permeability or non‐lysosomal release.
Drug efflux pumps	Check for upregulation of ATP‐binding cassette transporters (e.g., MDR1/P‐glycoprotein, BCRP) via gene or protein assays. Fluorescent substrate efflux assays (e.g., rhodamine‐123 or calcein‐AM accumulation assays).	Choose payloads that are poor substrates for efflux transporters or co‐administer efflux pump inhibitors.
Mutations in the molecular target of the payload	Sequence payload target genes (e.g., TOP1, tubulin isoforms) in post‐treatment samples. Longitudinal ctDNA analysis to detect emerging payload‐resistance mutations.	Switch to an ADC with a different cytotoxic mechanism or payload class.
DNA repair or survival pathway upregulation	Evaluate tumor expression of DNA damage–response genes and anti‐apoptotic factors. Phosphoproteomic profiling of ATR–CHK1 checkpoint activation with parallel evaluation of PARylation or DNA repair activity.	Rational combinations with DDR inhibitors or apoptosis‐sensitizing agents to disable tumor defense mechanisms. Exploit synthetic lethality combinations (e.g., ATR, PARP, or WEE1 inhibition).
Tumor microenvironment barriers	Utilize histological and imaging assessments of the TME to identify physical or biochemical barriers, such as dense stromal fibrosis, abnormal vasculature, or hypoxic regions. Spatial immune profiling to identify immune‐excluded vs. inflamed niches. Quantify Fcγ receptor–mediated ADC uptake by stromal macrophages.	Remodel the TME or improve drug delivery. Enzymatic degradation of stromal components (e.g., hyaluronidase) to break down the hyaluronic acid matrix. Select ADCs with strong bystander effects for poorly penetrated regions. Combine with stromal‐modulating agents (FAK, TGF‐β, CSF1R inhibitors).
Clonal evolution and plasticity	Perform serial biopsies or liquid biopsy (ctDNA) analyses over time to monitor tumor genetic evolution and phenotypic shifts. Single‐cell lineage tracing to map resistant cell‐state transitions. Longitudinal monitoring of antigen expression to detect target switching.	Adapt therapy in response to evolving tumor clonality. Deploy personalized multi‐target strategies (ADC cocktails or bispecific ADCs). Ecosystem‐guided ADC sequencing to maintain pressure on adaptive clones.
Epigenetic reprogramming	Utilize genomic profiling to identify tumors that enter drug‐tolerant states through epigenetic changes. Chromatin accessibility (ATAC‐seq) to detect drug‐tolerant persister states.	Epigenetic modulators can be employed to reverse these adaptations. Re‐sensitization strategies prior to re‐challenging with ADCs.

## 5 Linker Design and Immune Activation: A New Direction

### 5.1 Recent Trends and Innovations in ADC Linker Chemistry

Over the past five years, the overarching trend in ADC linker design has been a shift toward controlled release, with an emphasis on simultaneously maximizing plasma stability and enabling efficient, tumor‐selective payload liberation, while systematically mitigating unfavorable physicochemical properties such as hydrophobicity and aggregation.

A long‐standing challenge in ADC development has been the discrepancy between human and rodent plasma stability, particularly due to carboxylesterase activity (e.g., Ces1C). Although Val–Cit dipeptide linkers are relatively stable in human serum, premature cleavage in rodent plasma has limited preclinical translation. To address this limitation, P3‐modified tripeptide sequences incorporating acidic residues (e.g., Glu–Val–Cit) were introduced to suppress rodent plasma cleavage while preserving lysosomal processing, and have since become part of the standard linker design toolbox [124]. More recently, exo‐linker architectures, in which the cleavable peptide motif is structurally repositioned and combined with hydrophilic residues, have been reported to reduce premature payload release, aggregation, and enzyme‐mediated off‐target cleavage while enabling higher drug‐to‐antibody ratios (DARs) [125]. Beyond single‐enzyme cleavage, linker designs requiring sequential enzymatic processing for payload liberation have also emerged. For example, sulfonated glycoside‐based linkers require consecutive cleavage by arylsulfatase A followed by β‐galactosidase before payload release, effectively functioning as an AND‐gate–like mechanism [126]. Together, these strategies reflect a broader shift away from a cathepsin‐centric paradigm toward leveraging diverse tumor‐associated enzymatic programs to achieve greater biological selectivity and translational predictability.

As payload hydrophobicity increases, higher DARs often exacerbate aggregation, accelerate clearance, and shorten circulation half‐life—an issue repeatedly highlighted in analyses of approved ADCs. To mitigate these effects, linker chemistry has increasingly been leveraged as a physicochemical engineering tool. Polysarcosine (PSAR)‐based linker–drug platforms have demonstrated antibody‐like pharmacokinetics even at high DARs (e.g., DAR 8), effectively masking payload hydrophobicity, illustrating how linker composition can modulate ADC behavior independently of antibody or payload selection [127].

Although distinct from the cleavable linker motif, instability at the antibody–linker junction can also drive premature payload loss. Classical maleimide–cysteine conjugation is susceptible to retro‐Michael deconjugation, which can be mitigated through maleimide ring hydrolysis, thereby improving conjugate stability [128]. In parallel, maleimide alternatives such as bromoacetamide‐based chemistries have been proposed to achieve more stable cysteine attachment and improved ADC homogeneity [129].

Finally, AI‐driven linker engineering is emerging as a transformative force in ADC development. Machine learning and predictive modeling approaches are increasingly used to forecast linker and conjugation stability, and optimal conjugation sites, substantially accelerating design–build–test cycles [130]. Together, these advances underscore a paradigm shift in which linker chemistry is no longer viewed as a passive connector but rather as a tunable, programmable determinant of ADC pharmacology, safety, and therapeutic index.

### 5.2 Leveraging PROTAC Linker Design Principles to Inform Next‐Generation ADC Linker Engineering

Although ADCs and proteolysis‐targeting chimeras (PROTACs) are mechanistically distinct therapeutic modalities, recent advances in PROTAC linker engineering offer valuable conceptual insights that can be productively translated into ADC linker design. In PROTACs, linkers are not passive connectors but active determinants of biological function, governing ternary complex formation through precise control of linker length, flexibility, rigidity, and polarity [131]. This emphasis on linker geometry as a functional variable is directly relevant to ADCs, where linker architecture similarly influences enzymatic accessibility, intracellular processing, and physicochemical behavior, thereby shaping payload delivery efficiency rather than merely connecting molecular components.

Several emerging ADC linker strategies already reflect principles long established in PROTAC development. For example, exo‐linker and branched linker architectures conceptually parallel PROTAC‐inspired efforts to spatially reorganize linker components to optimize functional outcomes, such as reducing premature cleavage while preserving efficient intracellular release [125, 132, 133]. Likewise, the PROTAC field's systematic exploration of semi‐rigid and polarity‐distributed linkers provides a useful framework for addressing ADC‐specific challenges, including aggregation and pharmacokinetic liabilities associated with high DARs [134, 135]. Rather than relying solely on bulk hydrophilic masking, future ADC linkers may increasingly adopt distributed polarity and controlled rigidity to fine‐tune surface exposure and enzymatic engagement, treating linker composition as an adjustable biophysical parameter.

Importantly, PROTAC development has normalized the use of linker‐focused structure–activity relationship (SAR) campaigns, in which libraries of linkers are iteratively evaluated while the target‐binding components remain unchanged [136]. Applying a similar philosophy to ADCs—where linker length, branching geometry, and rigidity are systematically varied under fixed payload and conjugation conditions—could accelerate rational linker optimization beyond empiric trial‐and‐error approaches. This paradigm is further reinforced by the growing adoption of computational and AI‐guided modeling in PROTAC design, which enables multi‐parameter optimization of linker stability, polarity, and spatial orientation [137]. Such approaches are increasingly applicable to ADCs, particularly for predicting aggregation propensity, enzymatic accessibility, and developability at higher DARs [138].

Together, these converging trends suggest that while ADC and PROTAC linkers serve different immediate purposes—conditional payload release versus proximity‐driven degradation—the design logic underpinning PROTAC linkers offers a transferable blueprint for next‐generation ADC linker engineering. By embracing linker geometry, rigidity, and polarity as programmable variables, ADC development may further evolve toward more predictable, robust, and multifunctional therapeutic platforms.

### 5.3 ADC‐Induced Immune Activation Beyond Cytotoxicity

ADCs have long been developed as targeted cytotoxic agents to eliminate antigen‐expressing tumor cells. However, ADCs are increasingly recognized as multifunctional therapeutics capable of actively reshaping the TME and promoting antitumor immunity. This conceptual shift has been enabled by advances in cleavable linker design, payload chemistry, and higher DARs, which collectively extend ADC activity from direct tumor cell killing to immune engagement.

A broad range of ADC payloads—including auristatins, PBDs, and topoisomerase I inhibitors—can induce ICD following intracellular release via cleavable linkers. Regardless of payload class, ADC‐mediated cytotoxic stress converges on shared immunogenic outputs, including DAMP exposure and activation of innate immune sensing pathways. Notably, trastuzumab deruxtecan (T‐DXd) activates the cGAS–STING–type I interferon axis, resulting in dendritic cell activation and enhanced CD8^+^ T‐cell function [139]. These findings position cytotoxic ADCs as in situ immune‐priming agents that convert tumor cell death into a coordinated immune response.

In parallel, ADC‐induced immune activation can arise through direct remodeling of immunosuppressive cellular compartments within the TME. Immune‐modulating ADCs (IM‐ADCs), exemplified by CD25‐targeted conjugates, selectively deplete intratumoral regulatory T cells, thereby increasing effector CD8^+^ T‐cell infiltration, establishing durable antitumor immunity, and conferring protection against tumor rechallenge in preclinical models [140]. Importantly, these effects occur independently of immune checkpoint blockade, underscoring the capacity of ADCs to rebalance immune composition through spatially targeted cytotoxicity.

High‐DAR ADCs further amplify these immune effects by expanding intratumoral payload distribution and enhancing bystander killing. Although systematic comparisons across DARs remain limited, translational studies of high‐DAR ADCs such as T‐DXd reveal increased CD8^+^ T‐cell infiltration and immune gene signatures in responding tumors [141], suggesting that payload density can modulate the spatial reach and immunologic consequences of tumor cell death.

Insights gained from these immune‐activating properties of conventional cytotoxic ADCs have directly informed the development of deliberately immunostimulatory ADC architectures. Innate immune modulators activate dendritic cells and macrophages through pathways such as TLR and STING signaling, promoting antigen presentation and type I interferon responses. Immune‐stimulating antibody conjugates (ISACs) harness this biology by coupling innate immune agonists—most commonly TLR7/8 or STING agonists—to tumor‐targeting antibodies, thereby converting ADCs from passive delivery vehicles into localized immune activators while minimizing systemic toxicity.

In immunologically “cold” tumors such as breast cancer, ISACs represent a rational extension of ADC‐driven immune activation. HER2‐targeted ISACs have demonstrated robust myeloid activation and tumor regression in preclinical breast cancer models [142, 143], while BDC‐1001 has shown manageable safety and early clinical activity in HER2‐positive tumors, even in heavily pretreated patients [144]. Nevertheless, inflammatory toxicities observed with early ISAC candidates highlight the need for careful optimization of dose, linker stability, and immune agonist potency.

Collectively, these advances signal a transition from empiric cytotoxic ADC development toward intentionally immuno‐engineered ADC platforms designed to integrate tumor targeting with immune activation. Rather than serving solely as partners for immune checkpoint inhibitors, next‐generation ADCs may intrinsically integrate immunogenic payload release, immune‐modulating conjugates, and optimized DARs.

## 6 Future Directions and Clinical Translation

**FIGURE 4:**
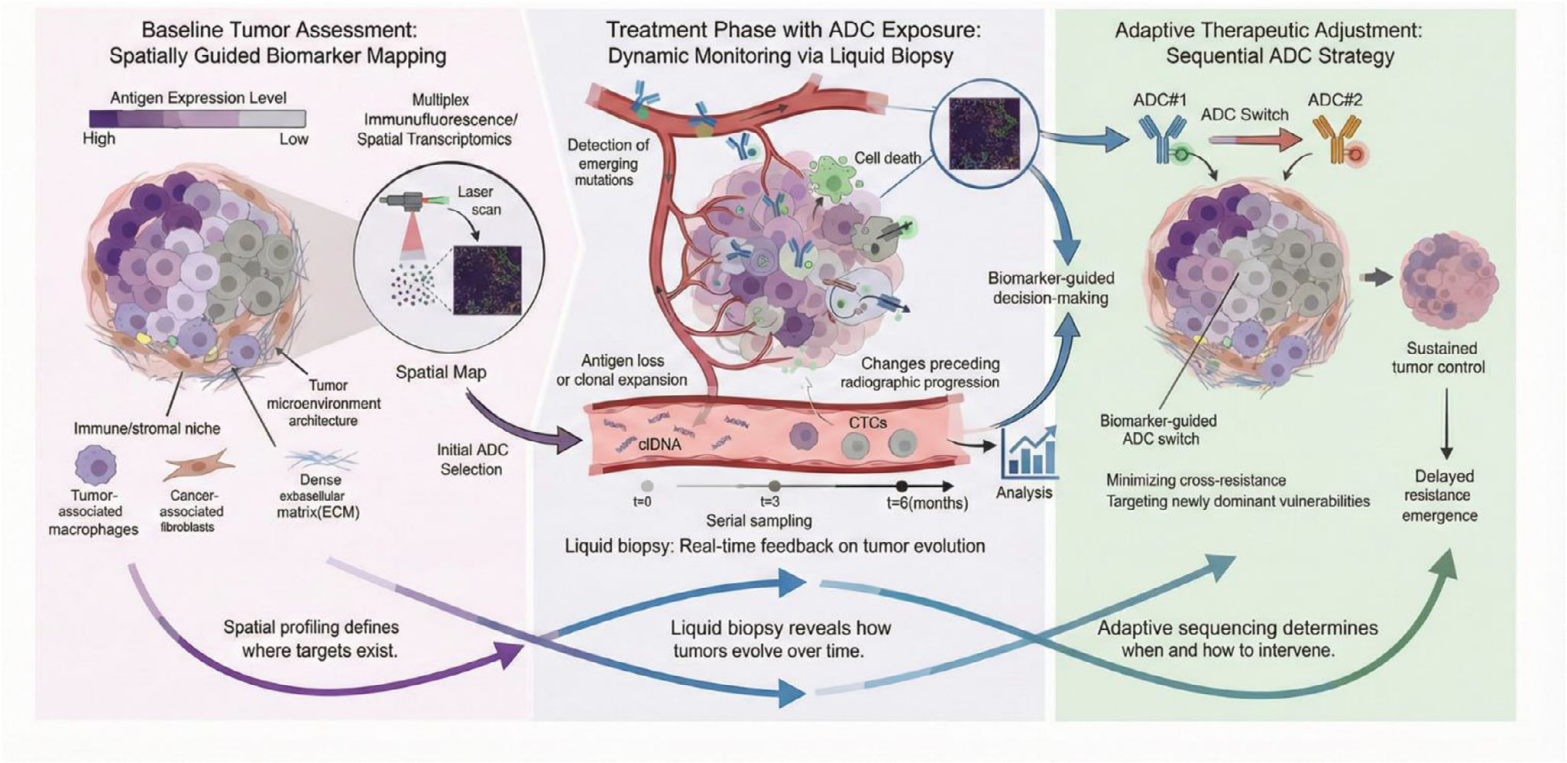
Integrated framework/strategy for overcoming ADC resistance through spatial profiling, liquid biopsy monitoring, and adaptive ADC sequencing. Baseline spatial profiling maps antigen distribution and tumor microenvironment features to guide initial ADC selection. During treatment, longitudinal liquid biopsy (e.g., ctDNA and circulating tumor cells) enables dynamic monitoring of tumor evolution, including antigen loss, clonal expansion, and emerging resistance. These real‐time insights support biomarker‐guided therapeutic adjustments, allowing sequential ADC switching to target newly dominant vulnerabilities, minimize cross‐resistance, and prolong tumor control.

In breast cancer, the sequential use of multiple ADCs has revealed diverse resistance mechanisms, including antigen loss or downregulation, impaired ADC binding, altered intracellular trafficking, and the activation of efflux pumps or bypass survival pathways. Collectively, these findings highlight the need for a systems‐level understanding of tumor ecosystems to effectively address therapeutic resistance. Such a framework recognizes spatial heterogeneity, clonal evolution, and microenvironmental dynamics as central drivers of treatment failure. Future efforts should therefore prioritize spatially informed biomarker profiling, liquid biopsy‐based real‐time monitoring, and adaptive sequencing of ADCs aligned with evolving tumor biology. As shown in Figure 4, integrating baseline spatial characterization with longitudinal liquid biopsy monitoring enables adaptive therapeutic adjustments to delay resistance and sustain tumor control.

### 6.1 Spatially Guided Biomarker Strategies

Spatially guided biomarker strategies offer a refined approach to capturing intratumoral heterogeneity and optimizing ADC therapy. Conventional bulk assays often overlook spatial context, including antigen‐negative regions and distinct immune niches that can affect drug efficacy. Advanced technologies, such as multiplex immunofluorescence and spatial transcriptomics, enable high‐resolution in situ mapping of antigen distribution, resistant subclones, and microenvironmental architecture, revealing the spatial organization of target antigens and resistance‐associated factors. This is especially relevant for ADCs that rely on bystander effects or require co‐expression of multiple targets, where the proximity of different cell populations influences therapeutic reach. By incorporating spatial biomarkers with computational and AI‐driven analytics, these approaches can move beyond descriptive profiling toward predictive modeling of ADC response, enabling patient stratification based on tumor architecture rather than single‐marker expression.

### 6.2 Liquid Biopsies for Real‐Time Resistance Monitoring

Liquid biopsies provide a complementary, dynamic view of tumor evolution during ADC therapy by capturing circulating tumor DNA (ctDNA) and circulating tumor cells (CTCs). Unlike serial tissue biopsies, which are invasive and spatially limited, liquid biopsies enable longitudinal assessment across multiple metastatic sites. Serial ctDNA profiling can identify resistance‐associated alterations, including antigen loss, target mutation, or clonal expansion, often preceding radiographic progression.

This temporal resolution allows clinicians to detect molecular escape earlier than conventional imaging and to adjust therapy before overt clinical failure, transforming resistance monitoring from a reactive to a proactive process.

### 6.3 Sequential ADC Design Informed by Ecosystem Adaptation

Sequential ADC strategies informed by tumor ecosystem adaptation represent a proactive framework to overcome resistance. Rather than viewing ADCs as single‐use therapies, this approach anticipates tumor evolution and strategically sequences agents in response to treatment‐induced biological changes. Clinical observations in metastatic breast cancer suggest that switching to ADCs with distinct payloads or alternative targets after progression can prolong disease control, supporting the concept that resistance is frequently payload‐ or antigen‐specific. Tumors that downregulate an initial target may remain susceptible to ADCs targeting alternative antigens, such as TROP‐2 or LIV‐1.

However, as multiple ADCs with overlapping targets and payload classes enter clinical practice, determining the optimal sequence has become increasingly complex, highlighting the need for biomarker‐guided decision frameworks rather than empiric selection. Resistance to one ADC may arise from antigen modulation, payload‐specific tolerance, or altered intracellular processing, each of which has distinct implications for selecting subsequent ADCs targeting alternative antigens or employing different cytotoxic mechanisms.

Operationally, this strategy integrates spatial profiling and liquid biopsy insights to guide ADC transitions. By aligning treatment selection with clonal shifts, antigen redistribution, and microenvironmental remodeling, clinicians can sustain therapeutic pressure on evolving tumors while minimizing cross‐resistance.

### 6.4 Personalized ADC Selection Guided by Tumor Context and Evolution

Personalized ADC selection represents a critical next step in translating advances in ADC engineering into meaningful clinical benefit. By integrating next‐generation ADC design with spatial biomarker profiling and real‐time resistance monitoring, ADC choice can be actively tailored to the evolving biological context of each patient's tumor. Rather than relying solely on baseline antigen expression, this strategy recognizes that therapeutic response is dynamically shaped by intratumoral heterogeneity, microenvironmental architecture, and treatment‐induced adaptation.

Spatial profiling identifies where therapeutic targets and resistant niches reside within tumors, while liquid biopsy‐based monitoring captures the temporal evolution of these features during treatment. Together, these data enable biomarker‐guided ADC selection and sequencing, allowing clinicians to deploy agents that match newly emerging tumor vulnerabilities as the disease adapts.

By framing ADC selection as a dynamic, continuously informed process, this model shifts ADC therapy from a static, one‐time decision to an adaptive strategy aligned with tumor evolution. Such personalization has the potential to delay resistance, refine sequencing decisions, and more effectively leverage the expanding ADC arsenal to achieve durable clinical responses.

## 7 Conclusion

ADCs have significantly reshaped the therapeutic landscape for breast cancer, offering potent, targeted activity where traditional therapies often fall short. Yet, the clinical impact of ADCs is frequently constrained by resistance mechanisms arising from tumor‐intrinsic factors, the TME, and dynamic host responses. To overcome these barriers, we must shift from reductionist, single‐target approaches to ecosystem‐level strategies that consider the tumor, stroma, and immune system as an interconnected network that influences treatment outcomes.

Current evidence suggests that sustained antigen expression, efficient payload delivery, and immune system engagement are all crucial for ADC success—each representing both a potential resistance point and a therapeutic opportunity. Thus, the future of ADC therapy lies in innovative design—embracing dual‐payload and bispecific constructs, as well as immune‐modulating ADCs—and in rational, biomarker‐guided combination regimens that target multiple elements of the tumor eco‐system simultaneously.

Furthermore, achieving durable control or a potential cure for metastatic breast cancer will require an integrated approach: leveraging advanced technologies to monitor resistance, designing inclusive clinical trials, and fostering collaboration across scientific and clinical communities. Through these efforts, we can realize the full potential of ADCs and move closer to transforming even the most treatment‐refractory breast cancers into controllable or curable diseases.

## Funding

The author have nothing to report.

## Conflicts of Interest

The authors declare no conflicts of interest.

## Data Availability

The authors have nothing to report.
